# Case of Lacerated Wound of the Abdomen

**Published:** 1873-05-15

**Authors:** Charles H. Norred


### CASE OF LACERATED WOUND OF ABDOMEN.

BY DR. CHARLES H. NORRED.

Mrs. M., aged 26, mother of two children, and in the eighth month of gestation, was gored by a cow, the horn entering the abdomen in the linea alba, two inches below the umbilicus, penetrating to the peritoneum, and producing laceration on each side, extending to crest of ilium. I saw her thirty minutes after the accident, and found her in a condition of collapse from shock; hæmorrhage slight; edges of wound everted, with portions of cellular tissue protruding from the margin. The latter were removed, and the wound closed by the continuous suture and adhesive straps. The sutures were passed deeply, almost to peritoneal surface; but in consequence of extensive sloughing it was found necessary to reapply them. I prescribed p. Doveri, ten grs., to be given as often as required when suffering, and to control uterine tenesmus, which was so severe as to seriously threaten premature labor.

On calling next day, found the bowels tympanitic, abdominal surface tender, pulse small and quick—120—and tongue dry and furred. I prescribed hyd. submur. et opii, every three hours, which resulted in a gradual cessation of inflammatory symptoms, the pulse falling to 80, and continuing so till convalescence was assured.

Twelfth day following the injury she was delivered of a healthy child, after a natural labor.

